# Ameloblastic Carcinoma: A Case Report with Emphasis on Relevant Diagnostic Aspects

**DOI:** 10.7759/cureus.111551

**Published:** 2026-06-26

**Authors:** Snehanjan Sarangi, Debarati Ray, Tathagata Bhattacharjee

**Affiliations:** 1 Oral and Maxillofacial Pathology, Dr. R. Ahmed Dental College and Hospital, Kolkata, IND

**Keywords:** ameloblastic carcinoma, cytological atypia, immunohistochemical, malignant transformation, metastasis

## Abstract

Ameloblastic carcinoma (AC) is a highly aggressive malignant odontogenic epithelial tumor. It may occur in a pre-existing ameloblastoma, odontogenic cyst, or de novo. The condition is typically painful, highly invasive, and multifocally metastasizing in nature, with the lungs being the most common secondary tumor site. The condition is most often encountered in adult men during the sixth decade, with a propensity towards the posterior mandible. Radiographic examinations typically exhibit ill-defined and aggressive lesions with massive bone loss. Histopathological examination shows classic cytonuclear atypia in the ameloblastic element. The immunohistochemical staining is generally found to be intense in markers such as Ki-67, p53, and p63. The majority of cases also carry mutations in the BRAF V600E gene. In this case report, we discuss the presentation pertaining to AC in a 29-year-old male patient, with an emphasis on the relevant clinical-radiological-pathological diagnostic aspects. The primary treatment of AC is surgical removal, occasionally in conjunction with dissection of the neck or radiation therapy to manage local and metastatic spread. In our case, surgical removal was performed. AC is a rare, aggressive, and invasive odontogenic epithelial malignant neoplasm. Further future studies regarding its pathogenesis, molecular genetics, and immunohistochemical aspects will help researchers in better understanding this novel entity.

## Introduction

Odontogenic malignant neoplasms are quite infrequent in their occurrence. Ameloblastic carcinoma (AC) is a rare malignant odontogenic epithelial tumor, comprising 0.3%-3.5% of all odontogenic neoplasms [1]. The term "AC" was emphasized by Elzay in 1982 [2].

The term "malignant ameloblastoma" (MA) should be properly distinguished from AC. MA is used to depict a neoplasm, connoting histopathological features of benign ameloblastoma along with metastatic capabilities. Whilst AC denotes a tumor with frank histopathological features of malignancy. It may or may not exhibit metastasis [3]. The majority of the lesions arise de novo, although a few might originate from a pre-existing ameloblastoma or an odontogenic cyst, like a dentigerous cyst or odontogenic keratocyst [4, 5].

ACs usually manifest themselves as painful, expansile lesions of the jaws, which might grow rapidly and cause cortical perforation. Occasionally, they can be accompanied by paresthesia, ulceration, or trismus. It generally involves the posterior mandible. These tend to occur over a wide age range and show a slight male predominance [6, 7]. Radiologically, a well-defined unilocular or multilocular radiolucent destructive lesion can be seen, frequently associated with cortical expansion and perforation [6]. AC possesses a 30% recurrence rate and 22% metastatic propensity [8].

According to the current WHO classification of odontogenic tumors, AC is categorized as a malignant odontogenic epithelial neoplasm that combines the architectural features of ameloblastoma with overt cytological evidence of malignancy [9]. In recent years, advances in molecular pathology have provided further insights into its pathogenesis, with BRAF mutations emerging as one of the most frequently reported genetic alterations. These molecular findings may aid in diagnostic assessment and could potentially influence future targeted therapeutic strategies [10, 11]. Herein, we report a case pertaining to AC with relevant clinical-radiological-pathological diagnostic aspects.

## Case presentation

A 29-year-old male patient reported to the Department of Oral Pathology, Dr. R. Ahmed Dental College and Hospital, Kolkata, West Bengal, India, with a chief complaint of a painful swelling involving the lower right jaw for the past 10 months. The patient did not possess any deleterious oral habits (chewable tobacco, smoking, or alcoholism). The medical and family histories of the individual were noncontributory. The hematological assays (total count (TC), differential count (DC), bleeding time (BT), clotting time (CT), erythrocyte sedimentation rate (ESR), platelet, hemoglobin %, INR, and blood glucose (fasting and postprandial)) were within normal limits.

Extraoral examination revealed the presence of a bony hard swelling involving the lower right jaw. The overlying skin was normal in coloration without any rise in temperature, ulceration, or discharge (Figure 1). Intraoral analysis showed the presence of an expansible swelling, extending from the buccal vestibular aspect of the 44 to 48 regions. It possessed prominent bucco-lingual cortical expansion (Figures 2a-2b). On palpation, slight fluctuation was evident on the vestibular aspect of the 45 and 46 regions. The lingual cortical expansion was bony hard in consistency and occupied the lingual aspect of the 44, 45, and 46 regions. 

**Figure 1:**
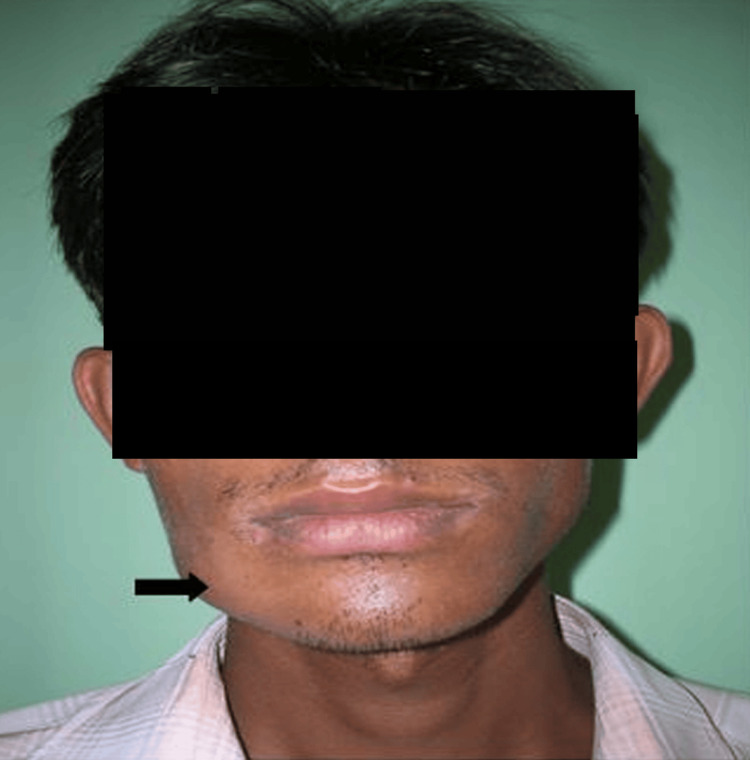
Extraoral examination revealed the presence of a bony hard swelling involving the lower right jaw (black arrow). Note: The patient provided written informed consent for the use of images for publication in an open-access journal.

**Figure 2:**
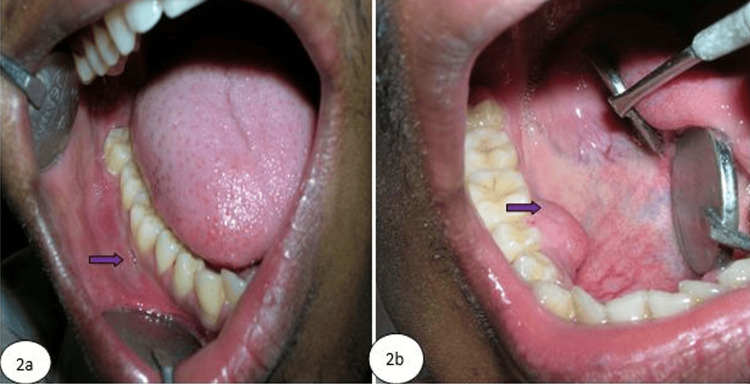
2a: Intraoral analysis showed the presence of an expansible swelling, extending from the buccal vestibular aspect of the 44 to 48 regions (purple arrow); 2b: The lingual cortical expansion was bony hard in consistency and occupied the lingual aspect of the 44, 45, and 46 regions (purple arrow).

The orthopantomograph (OPG) showed a fairly defined unilocular sclerotic radiolucency, expanding from 44 to 47 regions. Thinning of inferior cortices along with resorption of roots of 44, 45, 46, and 47 was evident (Figure 3).

**Figure 3:**
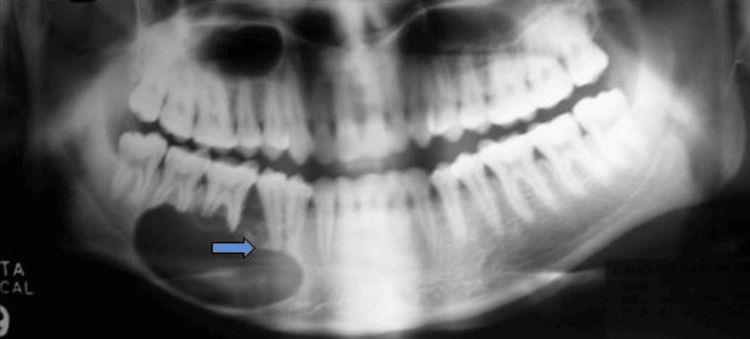
Orthopantomography (OPG) showed a fairly defined unilocular sclerotic radiolucency, expanding from 44 to 47 regions, along with thinning of inferior cortices alongside resorption of roots of 44, 45, 46, and 47 (blue arrow).

An incision biopsy was performed from the representative area, and the hematoxylin and eosin-stained sections showed the presence of fragmented zones of dysplastic stratified squamous epithelium, backed by fibrocollagenous connective tissue stroma. Within the stroma, follicles and interconnected strands of odontogenic epithelial cells were seen. The odontogenic epithelial islands and follicles were characterized by peripherally arranged intensely hyperchromatic and pleomorphic cells having marked cytonuclear atypia. Areas of keratin pearl formation were noted along with intermittent necrotic and hemorrhagic areas (Figures 4a-4d).

**Figure 4:**
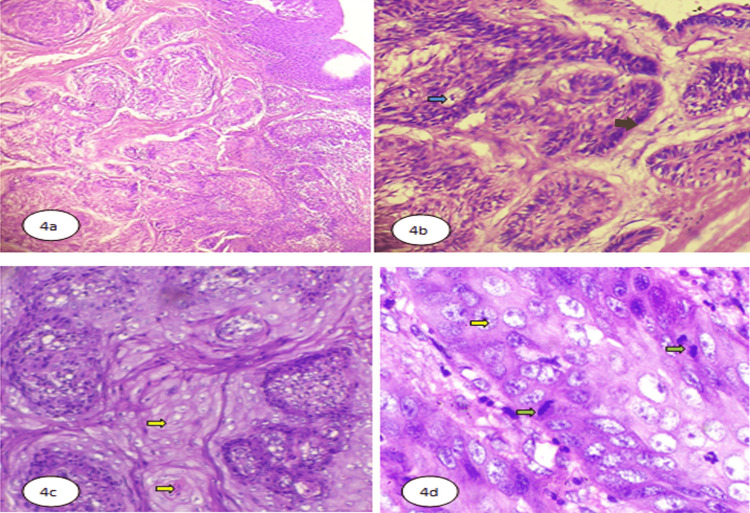
4a: Hematoxylin- and eosin-stained sections (4x magnification) showed the presence of fragmented zones of dysplastic stratified squamous epithelium, backed by fibrocollagenous connective tissue stroma bearing follicles and interconnected strands of odontogenic epithelium. 4b: (10x magnification) The odontogenic epithelial islands and follicles were characterized by peripherally arranged intensely hyperchromatic and pleomorphic cells (brown arrow) having marked cytonuclear atypia along with necrotic areas (blue arrow). 4c: (10x magnification) shows areas of keratin pearls (yellow arrow). 4d: (40x magnification) Atypical pleomorphic cells (yellow arrow) and mitotic figures (green arrow) are noted.

Immunohistochemical analyses revealed the presence of immuno-positivity of Ki-67 and p53 (Figures 5a-5b).

**Figure 5:**
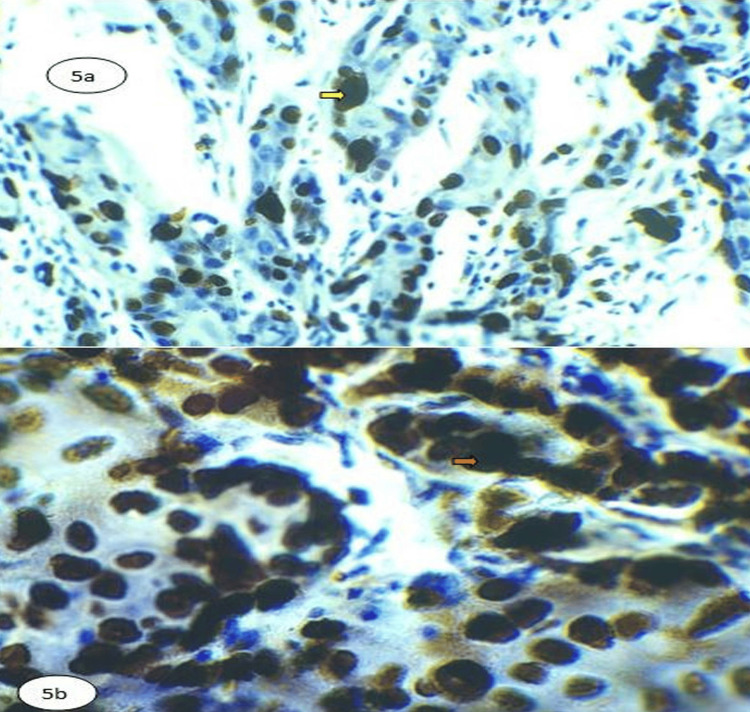
5a: Immunohistochemistry shows marked expression of Ki-67 (yellow arrow); Fig. 5b: Prominent p53 (orange arrow) expression is noted.

Based on overall clinical-radiological-microscopic-immunohistochemical features, a diagnosis of AC was made.

Surgical excision of the lesion was subsequently performed. Histopathological examination of the resection specimen pointed towards the diagnosis of AC. The patient was advised regular postoperative follow-up owing to the locally aggressive nature and metastatic potential of the lesion.

## Discussion

AC is a unique and hostile odontogenic epithelial malignancy. AC might arise from a pre-existing benign ameloblastoma that undergoes dedifferentiation or might occur de novo. The numerous lesions present within the spectrum of odontogenic carcinomas include AC, primary intraosseous carcinoma not otherwise specified, sclerosing odontogenic carcinoma, ghost cell odontogenic carcinoma, and clear cell odontogenic carcinoma [9]. 

The genetic variability landscape in AC is pretty diverse. BRAF V600E mutations are evident in many cases, with the prevalence ranging from 25%-40% [10]. Mutations pertaining to TP53, KRAS, APC, and CTNNB1 have also been observed along with epidermal growth factor receptor (EGFR) overexpression [11,12]. Somatic mutations affecting the Hedgehog pathway, specifically SMO, are quite evident [13]. Other genetic alterations associated with the occurrence of AC include RHOA, NLK, NFATC1, MMP7, DKK1, and CHSY1 [13]. Hypermethylation of p16 is associated with the malignant transformation of ameloblastoma towards AC [14]. The role of germline heterozygous mutations associated with FANCA p.S858R has been explored by Hurnik et al. [13]. 

AC usually tends to affect adult males during the 6th decade of life. The mandible is mostly involved, with an affinity towards the posterior mandible. The maxilla is affected less frequently [15]. These lesions are locally aggressive, invasive, large tissue masses that generally manifest as swellings along with ulcerations, significant bone resorption, tooth mobility, cortical expansion with occasional perforation, variable pain, paresthesia, trismus, etc. [16]. Metastasis is a significant feature of AC, and the metastatic sites include the lung, liver, bone, and brain. However, the lung is the commonest metastatic niche [13]. Cervical lymph nodes are the second most common affected site. Spread to the vertebrae, other bones, and viscera have also been noted. About 1% of all ameloblastomas tend to exhibit malignant transformation to AC [17].

AC radiographically shows aggressive, unilocular or multi-locular, ill-defined, destructive areas associated with cortical destruction. Effacement of the lamina dura, root resorption, or interspersed focal radio densities might be observed [18]. In our case, an OPG showed a fairly defined unilocular sclerotic radiolucency expanding from 44 to 47 regions, along with thinning of the lower cortical plates and regional tooth root resorption.

In the case of AC, the primary neoplasm or the metastatic deposits exhibit a microscopic pattern similar to an ameloblastoma, with the added cytological attributes of malignancy. The ameloblastomatous component displays features similar to those of follicular or plexiform ameloblastomas. The atypical characteristics include an increased nuclear-cytoplasmic ratio, hypercellularity, nuclear hyperchromatism, occasional perineural invasion, and the presence of enhanced mitotic figures. Necrosis in tumor islands and zones of dystrophic calcification might be noted. The lack of a central stellate reticular-like region and the presence of epithelial cell aggregates, cytosolic plexiform or insular, hyperchromatin, dense cellular arrangement, atypical nucleoli, focal necrosis, and neural and vascular invasion are considered important landmarks for the diagnosis of AC [1,2,6,19]. AC having a clear cell population tends to have a poorer prognosis along with increased tendencies for invasion, recurrence, and mortality [20].

There is another subtype, known as the spindled AC. It exhibits an ameloblastomatous epithelium with hypercellular stroma that shows elongated fibroblastic cells with scanty cytological atypia and scattered mitotic figures [21].

In stark contrast to AC, MAs show no microscopic features that differ from those of ameloblastomas, possessing a totally benign nature. This is evident with respect to the primary tumor site as well as the metastatic niches [15, 17].

In our case, within a fibrocollagenous stroma, follicles and interconnected strands of odontogenic epithelial cells were seen. The odontogenic epithelial islands and follicles were characterized by peripherally arranged intensely hyperchromatic and pleomorphic cells having marked cytonuclear atypia. Areas of keratin pearl formation and abnormal mitotic figures were noted along with intermittent necrotic and hemorrhagic areas.

In the present case, the diagnosis of AC was supported by the presence of characteristic ameloblastomatous architecture along with marked cytological atypia, increased mitotic activity, and focal necrosis. These features favored AC over atypical acanthomatous ameloblastoma, while the presence of odontogenic epithelial follicles and strands helped exclude primary intraosseous squamous cell carcinoma [1, 2, 19]. Additionally, the increased Ki-67 expression provided further evidence of the lesion's malignant potential [22-24].

Sometimes, there might be considerable overlap between the features of an aggressive ameloblastoma, such as a basaloid ameloblastoma, with that of AC. In those instances, immunohistochemistry plays an important role in distinguishing between an ameloblastoma and AC. Specifically, Ki-67, p53, and α-SMA are considered to be pertinent immunohistochemistry markers in delineating AC. Ki-67 and p53, which are tumor cell immune proliferative markers, display a strongly positive expression in cases of AC. As per Kase et al., a Ki-67 positivity index of 10% or higher is considered to be a diagnostic criterion for AC [22, 23]. In cases of AC, numerous other immunohistochemistry markers, such as p63, CK5, CK14, CK18, CK19, CD138, MMP-2 and 9, E-cadherin, SOX-2, and GPC-3, have been explored by other researchers [24-27].

In our case, a prominently high expression of both Ki-67 and p53 was also evident.

Treatment usually requires surgical resection with 2‐ to 3‐cm bony margins and contiguous neck dissection, both prophylactic and therapeutic. Postsurgical radiotherapy is also a consideration in many cases. The metastatic deposits in AC can be effectively managed by selective site radiotherapy and chemotherapy as necessitated [1, 28].

In nonmetastatic cases of AC, the five-year survival rate ranges from 69.1% to 83.2%, whereas in metastatic instances, it varies from 0% to 21.4% [29].

AC needs to be differentiated from lesions such as acanthomatous ameloblastoma, clear cell odontogenic carcinoma, MA, and primary intraosseous squamous cell carcinoma (PIOSCC). In acanthomatous ameloblastoma, peripheral palisading, reverse polarization, and stellate reticulum-like areas are evident with squamous metaplasia and keratinization. However, cytological features pertaining to malignancy, like cytonuclear pleomorphism, atypical mitoses, and necrosis, are not observed. In clear cell odontogenic carcinoma, the tumor is predominantly composed of nests and cords of clear cells, and it is characterized by EWSR mutation [30]. In MA, metastatic foci should be present, and these must not exhibit any cytological features of malignancy. In PIOSCC, the carcinoma component is comprised of moderately to poorly differentiated squamous epithelial cells, derived from odontogenic epithelium [2, 15].

## Conclusions

AC is a rare, aggressive, and invasive odontogenic epithelial malignancy. The present case highlights the importance of correlating the clinical and radiographic findings with the characteristic histopathological features of ameloblastic differentiation accompanied by cytological atypia and supportive immunohistochemical findings to arrive at an accurate diagnosis. Further studies exploring its molecular, genetic, and immunohistochemical aspects may enhance our understanding of this uncommon entity and contribute to the development of improved diagnostic and therapeutic approaches.
